# Protocol paper: multi-Centre randomised controlled trial evaluating a pre-clinic diabetes assessment and mapped care planning intervention amongst adults with type 1, type 2 or pre-diabetes

**DOI:** 10.1186/s13063-022-06475-7

**Published:** 2022-06-20

**Authors:** Ryan Charles Kelly, Peter Phiri, Hermione Price, Amar Ali, Irene Stratton, Kayleigh Austin, Alice Neave, Katharine Barnard-Kelly

**Affiliations:** 1 Spotlight Consultations Ltd, Portsmouth, UK; 2 Southern Health NHS Foundation Trust, Southampton, UK; 3 NHS Blackburn with Darwen CCG, Blackburn, UK; 4 Gloucestershire Hospitals NHS Foundation Trust, Gloucester, UK; 5 BHR Ltd, Fareham, UK

**Keywords:** Diabetes, Pre-clinic planning, Spotlight-AQ, Patient-professional communication, Randomised controlled trial

## Abstract

**Background:**

Existing therapeutic interventions to treat diabetes are well known, yet the majority of people with diabetes do not consistently achieve blood glucose targets (even individual therapy targets) for optimal health, despite the large range of treatment options available. Such outcomes have remained stubbornly poor for decades with <25% adults with diabetes achieving glycaemic targets. Patient behaviour, individually supported in routine clinical care, is an important missing component to improved outcomes, in a medical healthcare model not ideally suited to supporting successful diabetes management.

**Methods:**

A multi-centre, parallel group, individually randomised trial comparing consultation duration in adults with type 1, type 2 or pre-diabetes using the Spotlight Consultations pre-clinic assessment compared to usual care in the Spotlight-AQ study. Two hundred adults with type 1, type 2 or pre-diabetes attending routine care outpatient appointments across up to ten participating sites will be invited to participate.

**Intervention:**

An outpatient pre-clinic intervention delivered within 1 week prior to scheduled routine outpatient appointment.

**Primary outcome measure:**

Duration of routine outpatient consultation.

**Secondary outcome measures:**

- Functional health status
- Diabetes distress
- Depression
- Treatment satisfaction
- Impact on self-care behaviours
- HCP burnout
- HCP treatment satisfaction and burden
- Hypoglycaemia (time less than 70mg/dL)
- Hyperglycaemia (time above 180 mg/dL)
- Change in weight
- Change in HbA_1c_
- Cost effectiveness of intervention

**Discussion:**

Results from the study will provide valuable insights into patient-professional communication practices within routine care and recommendations will be made, as necessary, for improvements to that. If the intervention is shown to be clinically and cost-effective, the feedback from participants and healthcare professionals will be used to make any improvements prior to its deployment to support improved communication and associated health outcomes.

**Ethics and dissemination:**

The trial was approved by the Wales REC7 Research Ethics Committee (21/WA/0020). Results will be disseminated through national and international conferences, scientific journals, newsletters, magazines and social media. Target audiences include consultants and other clinicians in diabetes, and medical professionals or scientists overall.

**Trial registration:**

ISRCTN15511689. Registered on 10 November 2021

## Introduction

### Background and rationale

There is an urgent need to develop interventions that lead to sustained improvements in glycaemic and quality of life outcomes for people with diabetes and that support diabetes prevention. Therapeutic interventions to treat diabetes have been shown to be effective in clinical trials, yet >75% of people with diabetes consistently do not achieve blood glucose targets (even individual therapy targets) for optimal health, despite the large variety of drugs, including insulin, and medical devices available, e.g. insulin pumps [1]. Such glycaemic outcomes have remained stubbornly poor for decades and are a significant risk factor for microvascular and macrovascular outcomes. Patient behaviour, individually supported in routine clinical care, is an important missing component to improved outcomes, in a medical healthcare model poorly suited to supporting successful diabetes management.

As such, it is important to recognise the different needs of each individual and support skills development so that individuals are empowered to undertake effective disease self-management. Data from the International Diabetes Federation (IDF) shows that 10% of global health expenditure is spent on diabetes (US $760 billion), predicted to rise to $825bn by 2030 [2]. Indirect costs from premature death, disability and other health complications due to diabetes are estimated to add an additional 35% to the annual global health expenditure associated with the condition [2]. The intangible costs, however, are less visible but include worry, anxiety, discomfort, pain, loss of independence, concerns about managing the condition, fears for future complications and their potential impact on quality of life. These are also significant contributors to the cost of diabetes.

Depression is commonly reported to be 2–3 times more prevalent in people with diabetes than the general population [3]. This figure perhaps overshadows the significant number of individuals who do not report symptoms of depression, but experience diabetes distress. These emotions can be described as feeling overwhelmed and defeated by diabetes; feeling angry about diabetes, frustrated by the self-care regimen and/or having strong negative feelings about diabetes; feeling that diabetes is controlling their life; worrying about not taking care of diabetes well enough, yet unable, unmotivated or unwilling to change; avoiding diabetes-related tasks that give feedback about consequences of poor control; and/or feeling alone/isolated with diabetes. In type 2 diabetes (T2D), distress (but not depression) was related with poor glycaemic control and change in distress (but not change in depressive symptoms) was associated with both short- and long-term changes in glycaemic control [4]. Similar relationships were found in type 1 diabetes (T1D): diabetes-specific emotional distress (measured by the Diabetes Distress Scale) was related to glycaemic control in a Norwegian study and was also linked to worsening diabetes management over time in adults with T1D [5]. There are no existing standardised or validated strategies used in routine care to minimise the impacts of diabetes distress [6].

Routine patient care visits currently leave both patients and healthcare professionals feeling frustrated both in primary and specialist care settings. The lack of understanding of the psychosocial burden of diabetes and the evolving consequences results in a negative impact on clinical practice with consequential negative outcomes for patients and increasing frustration for healthcare professionals. Complex and detailed algorithms are supplied by various guidelines for the management of blood glucose, lipids, blood pressure and long-term complications, but these relate only to medical management. Even goals which have been mutually agreed upon are often not followed up, leaving patients frustrated and healthcare professionals struggling to provide tailored support. Typically, physicians interrupt their patients 11 s after they start describing their problems; approximately half of patients’ concerns are not discussed; and in half of health care visits, patients and physicians disagree on the central problem presented [7]. Disagreement about treatment goals, inconsistency amongst healthcare teams and confusion about treatment priorities are associated with poorer outcomes [6].

Burnout amongst healthcare professionals is a key challenge affecting healthcare practice, safety and quality of care. It is estimated that more than half of US physicians experience substantial symptoms of burnout, with burnout almost twice as prevalent amongst physicians as US workers in other fields [8]. Nurses experience a similarly high prevalence of burnout and depression, with 43% reporting high degrees of emotional exhaustion. COVID has exacerbated this problem. Furthermore, there are significant correlations between a physician’s sense of depersonalisation and patient satisfaction with their hospital care, and between a physician’s job satisfaction and patient satisfaction with their healthcare and patient-reported adherence to medical advice [8].

### Objective

The aim of the current study is to evaluate the clinical and cost-effectiveness of validated patient report outcome (PROs) pre-clinic assessment measures to identify patient priority concerns and mapped to evidence-based, theory-driven and resources to address those concerns. This addresses an urgent unmet need in routine care to improve communication and understanding for both people with diabetes and healthcare professionals.

## Trial design

Spotlight is an exploratory multicentre, parallel group, individually randomised RCT. Sites are primary and secondary care NHS centres located in Southampton, Wessex, Portsmouth, Bradford, Blackburn and South-West London. It will compare consultation length, biomedical and psychosocial outcomes in adults with type 1, type 2 or pre-diabetes attending routine outpatient appointments for their diabetes care. The number needed for a two-sample *t* test with standardised effect size of 0.50 at alpha=0.05, 90% power is 86 per group [9]. Thus, we will recruit a minimum of 100 participants for the control group and 100 participants for the intervention groups to allow for potential dropout. Previous feasibility data from real-world evidence collection has demonstrated feasibility, acceptability and improved consultations across three centres in the UK and USA [10].

## Study setting

The study will be conducted in primary and secondary care settings within England. A list of study sites can be found in the supplementary material.

## Eligibility criteria

### Inclusion criteria

- ≥18 years. There is no upper age limit.
- Diagnosed with T1D or at risk of or diagnosed with T2D (including pre-diabetes) for at least 6 months.
- Currently receiving any diabetes treatment.
- Willing/able to use Spotlight Consultations tool.
- Ability to give informed consent.
- Ability to speak and read English fluently.

### Exclusion criteria

- <18 years
- Lack of capacity

## Recruitment

### Screening and consent

Outpatient diabetes surgery appointment clinic lists will be scrutinised ahead of appointments and for people who appear eligible. An information sheet explaining the trial will be sent by post or email as appropriate (including contact details to opt out if the person does not want to be contacted about the trial). Potential participants who are approached have all consented to be contacted to discuss the study further. Before the outpatient appointment, a member of the research team will contact the prospective participant to discuss the study at least 24 h before the outpatient appointment allowing sufficient time for reflection and discussion. This will allow those who are eligible for the study to be randomised immediately after the outpatient appointment. If the participant wishes to take part, they will have the opportunity to discuss the study face-to-face with a research nurse before they give written consent. Final eligibility criteria will be checked before the participant is recruited. Patients whose medical records cannot be accessed prior to the appointment to determine eligibility (e.g. patients from another hospital) will be given information about the study on the day of their outpatient appointment and will be offered the opportunity to come on another day to discuss their participation in the trial. Research nurses at each participating centre will obtain informed consent from trial participants.

### Randomisation

Once the consent process has been completed, study participants will be randomised into either the intervention group or control group for 12 months. We will randomise on a 1:1 basis using computerised randomisation software. Sealed envelope online software randomisation will be used. Randomisation is blocked and stratified by centre (using random permuted blocks) to ensure groups are balanced.

Those randomised to the intervention group will be asked to complete study questionnaires every three months and the Spotlight Consultation pre-clinic assessment at baseline and follow-up scheduled routine outpatient appointments (Table 1). The results of the Spotlight Consultation digital health assessment will be discussed between the participant and healthcare professional during the outpatient visit along with identified matched care pathways and agreement made on best-fit action plan.

**Table 1:** Psychosocial questionnaires

Construct	Measure	Relibaility
Diabetes Distress	DDS [11]	*α*’s=.94
Depression	PHQ-2 [12]	*α*’s=.86
Anxiety	GAD-7 [13]	*r* = 0.782
Engagement	Self-Care Inventory (SCI) [14]	*R*=0.89
Treatment Satisfaction/Utility	Diabetes Treatment Satisfaction Questionnaire [15]	*a* = 0.92
Health Resource Utility	EQ5D [16]	*α*’s=.89
Well-being	WHO-5 [17]	*α*’s=.90
HCP Burnout	Maslach Burnout Inventory [18]	*α*’s=.90

All measures will be administered at baseline, 3, 6, 9 and 12 months

### Outcome measures

Primary outcome measure: duration of routine outpatient consultation.

Secondary outcome measures:

- Functional health status (EQ5D)
- Diabetes distress
- Depression
- Treatment satisfaction
- Impact on self-care behaviours (Self-Care Inventory)
- HCP burnout
- HCP treatment satisfaction and burden
- Hypoglycaemia (time less than 70mg/dL) (for those participants using CGM as standard care)
- Hyperglycaemia (time above 180 mg/dL) (for those participants using CGM as standard care)
- Change in weight
- Change in HbA_1c_
- Cost effectiveness of intervention (adapted CSRI and bespoke measures)

## The spotlight consultations intervention

### Intervention arm

Participants who are randomised to receive the Spotlight Consultations intervention will be sent a unique secure link to the Spotlight-AQ platform. The platform is commercially available and owned by Professor Barnard. The intervention consists of a validated pre-clinic questionnaire that provides insights into individual patient priority concerns across core domains of diabetes management. These priority concerns are presented graphically and form the basis of the discussion within the routine outpatient consultation. Mapped evidence-based resources are available to the HCP to share with their patient based on agreed goals set.

Telemedicine or in clinic visits: Participants will have a telemedicine or in-person clinic visit as per their usual routine care during the trial. Study participants will complete a personal assessment on study tablet devices if face-to-face visit. The support of a research assistant will be available if required. HCPs will also access the results and care pathway options, via their own secure portal logins. HCPs and participants will discuss the priorities and possible options collaboratively in a co-decision making, person-centred approach. Participants will be invited for repeat assessment as per routine care.

### Control arm

Participants in the control group will continue to receive usual care. Control group participants will be offered access to the Spotlight platform and pre-clinic assessment at the end of the RCT, for use in their next schedule routine outpatient appointment.

Implementing usual care appointments or the outpatient pre-clinic intervention (delivered within one week prior to scheduled routine outpatient appointment) will not change actual treatment for diabetic patients and these will continue for both trial arms.

### Follow-up

After the participants are randomised to either the intervention or control arm, data will be collected from them at the following timepoints:

- Pre scheduled routine outpatient appointment (baseline)
- Three, 6, 9 and 12 months post-randomisation

Participants will be sent reminders to complete follow-up questionnaires a week after they are due, as necessary.

There will be no special criteria for discontinuing or modifying allocated interventions.

Types of data that will be collected from medical records and questionnaire data, in addition to the baseline questionnaires that will be repeated, will be:

- Mortality
- Adverse events
- Health economic questions (adapted CSRI (client survey receipt inventory) questionnaire)

Healthcare professional questionnaire data (Maslach burnout inventory) will be collected at baseline, 6 and 12 months. A sub-group of six k healthcare professionals involved in the delivery of the intervention will be interviewed once around 12 months after the start of the trial in their centre.

The primary outcome data, i.e. length of each consultation, is collected using a stopwatch by participating healthcare professionals and recorded in the study records by the clinical trials team. All other outcome data is electronic and data analysts are not blinded; thus, unblinding will not occur.

### Qualitative evaluation

The aims of the qualitative evaluation are to understand and explore:

- Participants’ experiences, including gender-specific experiences, of receiving Spotlight Consultations tool and health professionals’ views about delivering it
- The perceived benefits of Spotlight Consultations from participants’ and health professionals’ perspectives, and their recommendations for future refinements
- Any changes participants make to their diabetes self-management practices and treatment goals after receiving Spotlight Consultations intervention and why
- Whether, in what ways and why, Spotlight Consultations is delivered and received differently in different settings
- Whether there are any site-specific differences in how participants self-manage their diabetes after receiving Spotlight Consultations, and why

#### Participant interviews

Twenty participants randomised to receive the intervention will be interviewed 6 months following their visit to explore whether, how and why, their diabetes self-management practices and treatment goals have changed in the intervening period, and any perceived barriers to achieving future changes and goals. These interviews will explore their experiences of receiving Spotlight Consultations tool; any changes made to their diabetes self-management practices, and why; short- and long-term treatment goals and the reasons for these; and perceived barriers and facilitators to achieving these goals. These interviews will also include detailed exploration of participants’ historical diabetes management practices; previous contact with health professionals and diabetes management programs; and their everyday work and family lives. The interviews will also explore participants’ information and support needs and whether, and in what ways, the intervention and follow-up care could be changed or improved. Purposive sampling strategies will be employed to ensure representation across age, duration of diabetes, type of diabetes/pre-diabetes, presence of complications and therapy. Consent will be taken as part of the overall informed consenting process.

#### Health professional interviews

Six health professionals involved in Spotlight Consultations delivery will be interviewed once at the end of the trial. Interviews will explore the following: previous experiences of delivering self-management interventions for adults with diabetes; perceived benefits of Spotlight Consultations as compared to other interventions; experiences of, and views about, the training received to deliver Spotlight Consultations; barriers and facilitators to intervention delivery; perceived impact of Spotlight Consultations on participants’ diabetes self-management practices; and how Spotlight Consultations could be changed/improved for future use. Consent will be taken as part of the overall informed consenting process. One healthcare professional will be randomly selected from each centre.

##### Qual topic guides

Participant and health professional interviews will be informed by topic guides, with questioning kept sufficiently flexible to enable individual issues to be identified and explored. Semi-structured interviews will be used with a combination of closed and open questions. All interviews will be audio-recorded, transcribed in full, and early interviews will be reviewed by the research team to determine whether any alterations to the topic guides need to be made.

#### Process evaluation

The process evaluation will be undertaken ‘to explain discrepancies between expected and observed outcomes, to understand how context influences outcomes, and to provide insights to aid implementation’. Interviews will be held with a subgroup of participants (*n*=6), healthcare professionals (*n*=3) and clinical triallists (*n*=2). These interviews will focus only on the experience of participating in the clinical trial, rather than questions about the intervention itself. The purpose of the process evaluation is to inform future roll-out of the intervention if proven to be clinically and cost-effective.

The study may be monitored, or audited in accordance with the current approved protocol, GCP, relevant regulations and standard operating procedures. All researchers involved in the study will have up to date GCP training. Adherence to intervention protocols will be reviewed periodically. The independent Trial Steering Group, consisting of clinical and psychosocial experts, PPI, statistician and trials manager will meet every six months to review trial conduct throughout the duration of the trial period.

##### Health economic analysis

Cost-effectiveness analyses to calculate quality-adjusted life years (QALYs) will be estimated from the EQ-5D-5L questionnaire and mortality data, using the area-under-the-curve method. A QALY is a measure of the state of health of a person, adjusted to reflect quality of life. One QALY equates to one year lived in perfect health [19]. Similarly, costs will be estimated at the patient level. Mean between-group differences in QALYs and costs will be estimated using a regression-based approach, including adjustment for baseline covariates and interaction terms for pre-defined sub-groups, and allowing for clustering at hospital and/or practitioner level. Results will be presented as an Incremental Cost-Effectiveness Ratio (ICER) if appropriate. Non-parametric bootstrapping will be used to estimate confidence intervals around estimated cost differences and ICERs.

A simple modelling approach will also be used to estimate the costs and health impacts of surgical complications over a lifetime horizon. This long extrapolation is necessary to reflect any mortality or lasting quality of life decrement associated with surgical complications. There will be no attempt to estimate the long-term impact of improved diabetes management related to the intervention, as it will be difficult to predict the duration over which any improvements will be maintained. This is likely to be a conservative assumption that will under-estimate the QALY gain and cost-effectiveness of intervention if it proves to be effective. Model parameters will be estimated from the trial and from other published sources.

## Data management plan

A data management plan (DMP) providing full details of the study-specific data management strategy for the trial will be available and a trial schedule with planned and actual milestones, CRF tracking and central monitoring for active trial management created. Data queries will either be automatically generated within the eCRF, or manually raised by the study team, if required. All alterations made to the eCRF will be visible via an audit trail which provides the identity of the person who made the change, plus the date and time.

At the end of the study after all queries have been resolved and the database frozen, the PI will confirm the data integrity by electronically signing all the eCRFs. The eCRFs will be archived according to Southern Health policy and a PDF copy including all clinical and Meta data returned to the PI for each participant. All data will be collected and stored in a secure password protected computers. Access to systems is severely restricted to specific research staff. Data collected during the course of the research will be kept strictly confidential and only accessed by authorised members of the trial team. All participants will be allocated an individual trial identification number and anonymised transcripts will be stored on a secure database. Audio recordings will be destroyed immediately following quality assessment of transcription.

Only clinical trials staff will have access to the final trial dataset for data cleaning and analyses.

## Statistics

Demographics and characteristics of participants at baseline will be summarised and assessed for comparability between the intervention and control arms [20]. The primary analysis will be conducted using ANCOVA adjusted for randomisation stratification factors on an intention to treat population. Continuous data will be presented as means and standard deviations and analysed using ANCOVA (or presented as medians and ranges and analysed using Mann-Whitney *U* tests if data are skewed). Binary data will be reported in terms of odds ratios and analysed using logistic regression modelling. Analysis of time-to-event outcomes will include presenting Kaplan-Meier graphs by arm and analysed using Cox proportional hazards regression (or competing risk regression as discussed below).

A two-sided *p*-value of 0.05 or less will be used to declare statistical significance for all analyses and results will be presented with 95% confidence intervals. Subgroups will be investigated, including those with HbA_1c_ above or below 69 mmol/mol at presentation, type of diabetes and age above or below 75 years. The cut-off of 69 mmol/mol has been chosen as the level above which the Joint British Diabetes Societies recommend specific action to improve pre-operative glycaemic control. Missing data will be imputed using an appropriate method, such as multiple imputation, in line with the statistical analysis plan. If a participant who has given informed consent decides during the course of the study to discontinue or withdraw their participation before the study period ends, they will be withdrawn from the study. Identifiable data already collected with consent would be retained and used in the study. No further data would be collected or any other research procedures carried out in relation to the participant. The reason for withdrawal will be recorded in the CRF.

## Potential risks

The risks associated in taking part are very small. Taking part may make participants think more about their own mood and how they feel about their diabetes, their approach to self-management and views on diabetes burden and its impact more broadly. The study team will make every effort to avoid compromising a participant’s confidentially that may result in serious negative social, legal or economic ramifications for the participant. The team will adhere to ethics regulations during this study.

### Adverse event reporting

Any and all untoward medical occurrence in a participant or clinical study participant, diabetes-related or otherwise, which does not necessarily have a causal relationship with study treatment or participation. An AE can therefore be any unfavourable and unintended sign (including an abnormal laboratory finding), symptom or disease temporally associated with the study treatment or participation (regardless of causality assessments). Serious adverse events are not anticipated due to the nature of the study.

Formal stopping rules do not apply as there are no anticipated problems that are detrimental to the participant.

## Data monitoring committee

A external data monitoring committee will be established to provide external independent oversight of the conduct of the clinical trial. This committee will meet every 6 months and consist of individuals from lay and multi-disciplinary teams.

## Strengths and limitations of the study

- *Novelty of the intervention / the fact that it addresses an important gap*
- *Choice of outcome measures will enable a complete evaluation of both clinical outcomes and cost-effectiveness*
- *Sample size and number of sites will make the results representative of a wide population and centres with differences in practice*
- *Intervention can be delivered face-to-face or remotely due to COVID and practical issues*
- *COVID-19 may create delays and recruitment problems*

- *Intervention requires use of tablet device (provided) however is unsuitable for adults with low literacy or poor English*

## Dissemination plan

Results of the study will be submitted for publication in peer reviewed journals and for presentation at national and international scientific conferences (American Diabetes Association, Advanced Therapeutics and Technologies in Diabetes, Diabetes UK Annual Professional Conference). Results will also be disseminated to trial participants via letter and more broadly via patient conferences and advocacy group meetings.

## Trial status

Protocol v.1.10; 24 March 2021

Recruitment start: 18 October 2021; recruitment completion: 31 March 2022

## Data Availability

On request from the PI.
